# Effects of Feeding Time on Markers of Muscle Metabolic Flexibility Following Acute Aerobic Exercise in Trained Mice Undergoing Time Restricted Feeding

**DOI:** 10.3390/nu13051717

**Published:** 2021-05-19

**Authors:** Aaron Persinger, Matthew Butawan, Martina Faietti, Ashley Pryke, Kyley Rose, Marie van der Merwe, Richard J. Bloomer, Melissa J. Puppa

**Affiliations:** College of Health Sciences Memphis, University of Memphis, Memphis, TN 38152, USA; ampersinger@gmail.com (A.P.); mbutawan@cbu.edu (M.B.); martina.faietti95@gmail.com (M.F.); prykeya@gmail.com (A.P.); krose28@uthsc.edu (K.R.); mvndrmrw@memphis.edu (M.v.d.M.); rbloomer@memphis.edu (R.J.B.)

**Keywords:** time-restricted feeding, aerobic training, skeletal muscle metabolism

## Abstract

Time-restricted feeding (TRF) is becoming a popular way of eating in physically active populations, despite a lack of research on metabolic and performance outcomes as they relate to the timing of food consumption in relation to the time of exercise. The purpose of this study was to determine if the timing of feeding/fasting after exercise training differently affects muscle metabolic flexibility and response to an acute bout of exercise. Male C57BL/6 mice were randomized to one of three groups for 8 weeks. The control had ad libitum access to food before and after exercise training. TRF-immediate had immediate access to food for 6 h following exercise training and the TRF-delayed group had access to food 5-h post exercise for 6 h. The timing of fasting did not impact performance in a run to fatigue despite TRF groups having lower hindlimb muscle mass. TRF-delayed had lower levels of muscle HSL mRNA expression and lower levels of PGC-1α expression but displayed no changes in electron transport chain enzymes. These results suggest that in young populations consuming a healthy diet and exercising, the timing of fasting may not substantially impact metabolic flexibility and running performance.

## 1. Introduction

Over the past five years, interest in intermittent fasting has dramatically increased. Internet searches are as much as 25% more popular for terms related to intermittent fasting compared to the peaks of the previous five years [1]. Unlike caloric restriction, where one restricts caloric intake but not the timing of consumption, intermittent fasting restricts only the time of consumption, resulting in extended fasting times. The duration of the fast can be as long as 24 h—this is termed alternate day fasting (ADF) [2], or only limited hours of a day, which is now commonly referred to as time-restricted feeding (TRF). During TRF, the fasting time can vary in length and can be as short as eight hours [3,4] or as long as 18 h [5].

Previous studies have shown that TRF results in a reduction in body weight as well as improvements in metabolic parameters, including reductions in total cholesterol, triglycerides, insulin, glucose, and inflammatory cytokines IL-6 and TNFα [6]. These previous studies point toward an improvement in metabolic flexibility when consuming a high-fat diet [3,4,7]. One mechanism by which TRF may be preventing metabolic dysfunction is through improved or preserved muscle mitochondrial function and mitochondrial biogenesis [8,9], as the protection provided by TRF resembles changes in skeletal muscle induced by exercise [10].

Muscle contraction regulates intracellular pathways that control mitochondrial function [11,12]. 5′ adenosine monophosphate-activated protein kinase (AMPK) and mammalian target of rapamycin (mTOR) play major roles in promoting metabolic flexibility and mitochondrial function through their interactions with the mitochondrial energy creation pathways, thus maintaining metabolic health and regulating the replenishment of energy stores post exercise [13,14]. TRF also induces the activation of AMPK, which has been shown to signal mitochondrial biogenesis and may be one mechanism for the metabolic benefits of both TRF and exercise [15,16,17].

The replenishment of used energy stores after exercise is theorized to be improved by the consumption of a meal within a certain time frame of completion [18]. Although the idea of a metabolic/anabolic window is mostly used in strength training, nutrient timing is commonly used with aerobic exercise as well [19]. Research is limited when it comes to aerobic exercise and is further complicated by the composition and timing of the diet. There is limited research indicating that exercising in a fed state leads to improved aerobic performance if the exercise is long enough. However, if the exercise time is short (~60 min or less), there is no significant difference in performance [20]. There is a lack of research on how the timing of a feeding window can affect aerobic performance and metabolic flexibility in a healthy population.

Therefore, the purpose of this study was to determine if the timing of feeding/fasting after exercise training differently affects muscle metabolic flexibility and response to an acute bout of exercise. We hypothesize that delaying feed time post training will result in decreased metabolic flexibility and markers of mitochondrial content compared to mice with access to food immediately post training or with ad libitum access to food.

## 2. Materials and Methods

### 2.1. Animals and Experimental Design

All animal experiments were approved by the University of Memphis IACUC (protocol #0833). Six-week-old C57BL/6 male mice (*n* = 36) were purchased from Envigo (Indianapolis, Indiana). Animals were housed in a USDA-approved animal facility on the University of Memphis campus. Upon arrival, all mice were acclimated for three weeks, during which time they were entrained to a reverse light cycle with lights off (active phase) from 6 a.m.–6 p.m. During this time, all mice had ad libitum access to food and water. Mice were housed two per cage. After 3 weeks, mice were randomly assigned their fasting and exercise routines and body mass was monitored twice weekly. Mice were divided into 3 groups, 12 mice per group, which differed in timing of food availability. Two groups followed a time-restricted feeding protocol where they had 6 h of food access and 18 h of fasting, with ad libitum access to water. The third group had ad libitum access to food 24 h a day. Mice were kept on their respective diet protocols for 8 weeks. Two mice died during initial testing, and 5 died from infighting and an acute infection. A vet examined the bodies and approved the continuation of the study. These mice were removed from the results. On the day of the sacrifice, the mice completed their standard exercise protocol of 60 min of running. Thirty minutes post completion, animals were sacrificed and hindlimb muscles including the gastrocnemius, soleus, plantaris, quadriceps, EDL, and tibialis anterior were collected, weighed, and placed in liquid nitrogen. They were stored at −80 °C until analysis.

### 2.2. Diet and Feeding Protocol

Mice were provided a growing rodent chow, which consisted of 21% protein, 15% fat, and 64% carbohydrate (AIN-93G, Research Diets, New Brunswick, NJ, USA) for the duration of the study. The TRF-delayed group had food access from 12:00 p.m. until 6:00 p.m.; food access was provided approximately 5 h following the cessation of exercise. The TRF-immediate group had food access from 8:00 a.m. until 2:00 p.m. Food access was given immediately post exercise. The control group had ad libitum access to food before and after exercise. Food was weighed daily, and the amount consumed per cage calculated. All mice were provided unlimited access to food during their respective feeding windows.

### 2.3. Exercise

All mice exercised 5 days a week for the 8 weeks. In the first week of exercise, all mice underwent a familiarization protocol. This familiarization protocol included the standard warm-up consisting of 5 min at 5 m/min, 5 min at 10 m/min and 5 min at 15 m/min at a 10% incline followed by 30 min at 20 m/min.

Starting at week 2 through the end of the study, all mice ran a protocol that consisted of the same 15-min warm-up. After this warm-up period, the mice ran for 45 min at 20 m/min for a total of 1 h running. All mice completed the exercise training within the first three hours of the active phase. The TRF-delayed group began running at 6:00 a.m., TRF-immediate started at 7:00 a.m., and the control group began running at 8:00 a.m. Upon completion of their exercise, mice were returned to their cage.

### 2.4. Run to Fatigue

After one week of familiarization with the treadmill (3/6 Open Treadmill, Columbus Instruments, Columbus, OH, USA), mice underwent a run to fatigue test to assess performance. The test was performed as follows: mice were allowed to warm up, and after 20 min at 20 m/min, the speed was increased to 25 m/min. The incline was set at 5%. The mice then ran until fatigue set in. Mice were deemed fatigued if they, upon nudging, were not able to continue running. Mice performed two run to fatigue tests, one immediately prior to the start of TRF protocols and the second at the end of week 8. Mice were given two days off between their last bout of exercise training and their run to fatigue.

### 2.5. Protein Expression

Protein was extracted from the gastrocnemius muscle. The tissue was homogenized on ice using Mueller Buffer containing protease and phosphatase inhibitors. The samples were centrifuged for ten minutes at 10,000 g’s at 4 °C. Supernatant was placed in a new tube and diluted with Diluent Buffer. Protein concentration was measured, and 20 µg of protein was loaded into polyacrylamide gradient gel (4–15%) and subsequently transferred onto a PVDF membrane. The transfer efficiency was determined by Ponceau staining. The membrane was blocked in 5% Bovine Serum Albumin in TBST for an hour. Primary antibodies were incubated overnight. The following were assessed: PAMPK and AMPK (Cell Signaling Technology, Danvers, MA, USA), PGC1α, CVATP5A, CIIUQCRC2, CIVMTC01, CIISDHB, and CINUUFB8—total OXPHOS (ABCAM, Cambridge, UK). All antibodies were used at a 1:2000 dilution in the 5% Bovine Serum Albumin in TBST. The next day, the membranes were placed in the appropriate secondary antibody for two hours, washed in TBST, and imaged using chemiluminescent agent in an iBright FL1500 imaging system (Thermo Fisher Scientific, Waltham, MA, USA). All samples were normalized to loading controls. ImageJ software was used to quantify the bands. All representative Western blot images for each protein come from the same membrane.

### 2.6. Gene Expression

RNA was extracted from the gastrocnemius using trizol (Ambion, Austin, TX, USA) following the manufacturer’s specifications. cDNA was prepared using a high-capacity cDNA kit (Applied Biosystems, Waltham, MA, USA). Expression of the following genes *Tfam*, *Pgc1α*, *Fabp*, *Cd36*, *Hsl*, *Crat*, *Pfk*, *Glut4*, *Fasn*, *Ldhb*, and *Gapdh* (Table 1) was performed using RT-PCR. GAPDH was used as a reference gene. Primers were ordered from IDT (Coralville, IA, USA). The PCR was run using PowerUp SYBER green (Applied Biosystems, Waltham, MA, USA). All PCR was run on a QuantStudio 6 instrument (Applied Biosystems, Waltham, MA, USA). Data were analyzed using the delta delta ct method.

### 2.7. Statistical Analysis

All data are presented as mean ± SEM. A repeated measures ANOVA was used to assess differences across time and conditions. A one-way ANOVA was used to compare across conditions. Tukey post hoc analysis was used to examine interactions. GraphPad Prism 8 (San Diego, CA, USA) was used to analyze all data. Statistical significance was noted at *p* < 0.05.

## 3. Results

### 3.1. Body Weight Changes

**Figure 1:**
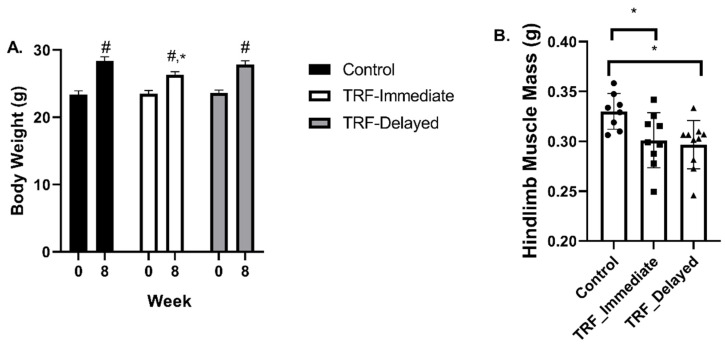
Body weight and muscle mass. (**A**) Body weight measured at the beginning and end of the 8-week study. (**B**) Hindlimb muscle mass consisting of the gastrocnemius, quadriceps, soleus, plantaris, tibialis anterior, and extensor digitorum longus was measured at the time of sacrifice. All muscles were weighed prior to being frozen in liquid nitrogen. All data are presented as mean ± SEM. A repeated measure ANOVA was used to identify differences in body weight over time. An ANOVA was used to measure changes in hindlimb mass. Significance was set at *p* ≤ 0.05. * significant compared to control at same timepoint. # significant compared to week 0 within group.

Body weight was measured at week 0 and week 8 (Figure 1A). There was an effect of time (*p* < 0.0001), suggesting that all mice continued to grow throughout the study. At week 8, the TRF-immediate group weighed an average of 2 g less than the control group (*p* = 0.017). All groups had a significant increase in body weight through the 8 weeks of the study. Hindlimb muscle mass was significantly smaller in both the TRF-immediate and the TRF-delayed group compared to the controls (*p* = 0.02), while there was no difference in the TRF-immediate group compared to the delayed group (Figure 1B). Despite significant differences in total hindlimb mass, there were no significant effects of TRF in the more glycolytic muscles including the EDL and the plantaris (Table 2). Additionally, there was no effect of feeding regimen on the overall body size measured by tibia length, *p* = 0.47 (Table 2).

### 3.2. Energy Status Following Acute Exercise in Chronic TRF with Immediate and Delayed Feeding

**Figure 2:**
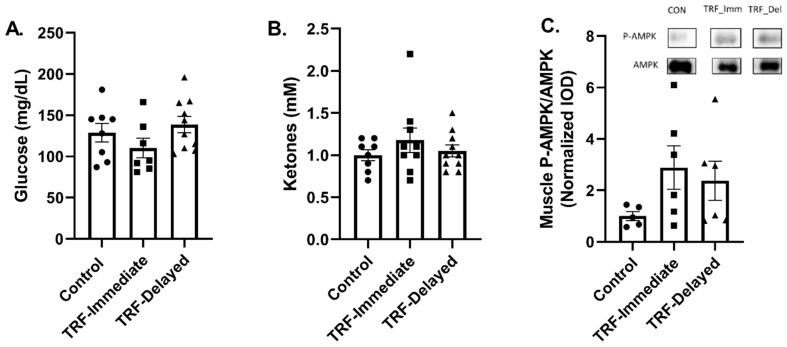
Markers of energy status in following acute exercise in trained mice with chronic TRF. Plasma (**A**) glucose and (**B**) ketones were measured immediately following completion of 60 min of aerobic exercise prior to sacrifice. (**C**) Skeletal muscle AMPK phosphorylation was measured in the gastrocnemius muscle and representative bands from the same blot are presented. Data are expressed as mean ± SEM. All data were analyzed with ANOVA and significance was set at *p* ≤ 0.05.

We next examined markers of energy status following an acute bout of exercise after eight weeks of chronic TRF and endurance training. No difference was seen in plasma glucose levels (Figure 2A) or in plasma ketone levels after exercise (Figure 2B). To assess if there were differences in levels of energy stress in the muscle after an acute bout of exercise, we measured the phosphorylation of AMPK, which is known to be activated in conditions of energy stress such as fasting and exercise. There was no significant difference in the level of AMPK phosphorylation with chronic TRF (Figure 2C); however, approximately half of the samples demonstrated higher AMPK phosphorylation with TRF. These data suggest that chronic time-restricted feeding may not alter the energy stress in skeletal muscle after an acute bout of exercise in healthy mice, but there is variability between subjects.

### 3.3. Markers of Metabolic Flexibility Following Acute Exercise in Chronic TRF with Immediate and Delayed Feeding Following Training

**Figure 3:**
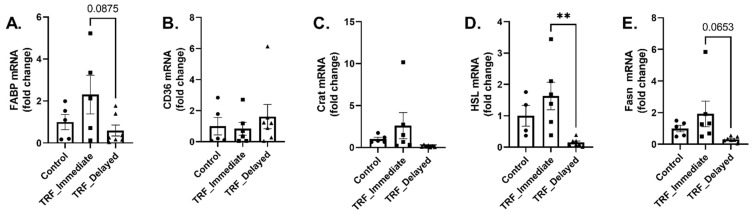
Transcriptional regulation of markers of lipid metabolism. mRNA expression of (**A**) FABP, (**B**) CD36/FAT, (**C**) Crat, (**D**) HSL, and (**E**) Fasn was measured in the gastrocnemius muscle. Data are presented as mean ± SEM. One-way ANOVA was used to detect significance. ** indicates significance of *p* ≤ 0.01.

Metabolic flexibility is the ability to change substrates based on availability and energy demands. We examined several markers of metabolic flexibility in the gastrocnemius muscle after an acute bout of exercise. The transcription of fatty acid binding protein (FABP) trended to be lower in the TRF-delayed mice compared to the TRF-immediate group (*p* = 0.08) (Figure 3A). There were no differences in mRNA levels of CD36/FAT (Figure 3B) or Crat (Figure 3C), which are involved with lipid metabolism and transport; however, there was a significant decrease in hormone sensitive lipase (HSL) mRNA levels (*p* = 0.006) (Figure 3D). Fatty acid synthase levels trended to be decreased in the TRF-delayed group (*p* = 0.07) (Figure 3E). This suggests that lipid metabolism may be lower after acute exercise in animals that chronically delay feeding after training; however, functional or enzymatic assays would be needed to measure this further.

**Figure 4:**
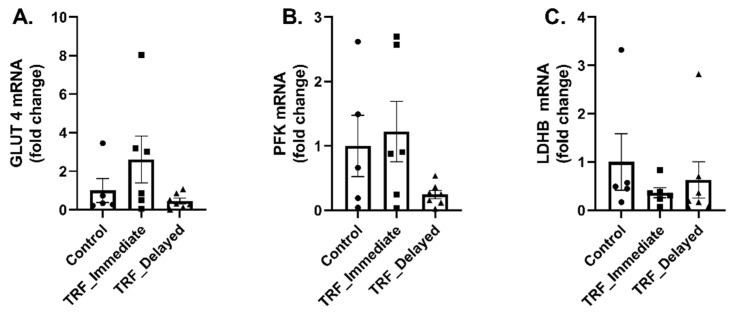
Transcriptional regulation of markers of glucose metabolism. mRNA expression of (**A**) GLUT4, (**B**) PFK, and (**C**) LDHB was measured in the gastrocnemius muscle. Data are presented as mean ± SEM. One-way ANOVA was used to detect significance.

To further examine the effects of acute exercise on metabolic flexibility in trained mice with altered feeding time during TRF, we measured the transcription of genes regulating glucose metabolism. There were no significant changes in the transcription of genes regulating glucose metabolism, including *Glut4* (Figure 4A), *Pfk* (Figure 4B), and *Ldhb* (Figure 4C).

### 3.4. Marker of Mitochondrial Biogenesis and Content

**Figure 5:**
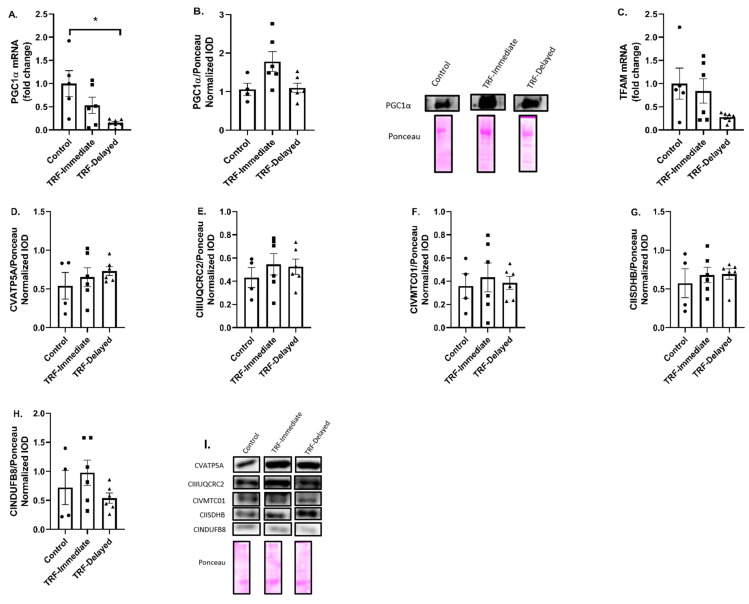
Markers of mitochondrial biogenesis and content after acute exercise in gastrocnemius of mice exposed to 8 weeks of training and TRF. (**A**) PGC-1α mRNA and (**B**) protein expression was measured in the gastrocnemius. (**C**) TFAM mRNA expression and (**D**) CVATP5A, (**E**) CIIUQCRC2, (**F**) CIVMTC01, (**G**) CIISDHB, (**H**) CINUUFB8 were measured in the gastrocnemius. (**I**) Representative Western blot images. All protein data were normalized to Ponceau stain loading control. Data are presented as mean ± SEM. One-way ANOVA was used to detect significance. * indicates significance *p* ≤ 0.05.

To better understand the changes in markers of lipid metabolism, we next examined markers of mitochondrial biogenesis and content. PGC1α mRNA expression was significantly lower in the gastrocnemius of the TRF-delayed compared to the control group (*p* = 0.008) (Figure 5A). PGC-1α protein levels were trending to be increased in the TRF-immediate group compared to both the control (*p* = 0.072) and the TRF-delayed (*p* = 0.057) groups (Figure 5B). TFAM activates the transcription of mitochondrial genes and is a known target of PGC1α. There were no significant differences in TFAM mRNA expression between groups in the gastrocnemius (Figure 2B); however, there was a trend towards a decrease in the TRF-delayed group’s gastrocnemius compared to the control (*p* = 0.087) (Figure 5C). There was no change in any markers of mitochondrial content with time-restricted feeding measured through protein expression of electron transport chain components (Figure 5D–I).

### 3.5. Run to Exhaustion

**Figure 6:**
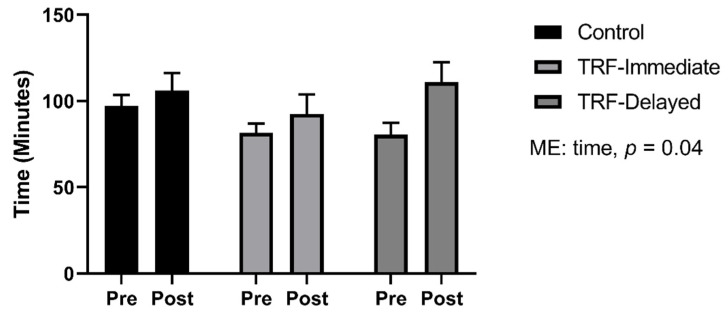
Run to exhaustion test was run both before and after 8 weeks of time-restricted feeding and exercise training. All data are presented as mean ± SEM. A repeated measures ANOVA was run to identify differences between groups and time.

We determined run to exhaustion time to see if overall performance was altered by TRF. Mice performed a run to exhaustion before and after 8 weeks of training. There was a main effect of time on performance (*p* = 0.04). The control group improved by 14% ± 43%. The TRF-immediate group increased by 13% ± 33%, and the TRF-delayed group improved by 55% ± 86%; however, there was no significant group effect, suggesting that the timing of feeding had no effect on performance in trained mice (Figure 6).

## 4. Discussion

Over the last few years, lifestyle modifications that restrict the timing of food have become popular methods to prevent metabolic disorders and control weight. In addition, some athletes utilize time-restricted feeding in an effort to enhance performance. There is limited evidence demonstrating the effect of the timing of fasting on the acute response of the muscle to an exercise stimulus in trained individuals who chronically follow a time-restricted feeding protocol. Therefore, we sought to examine the role of these lifestyle modifications on the acute response to exercise in young, healthy male mice.

In our study, TRF had no effect on body weight over the 8-week period; however, hindlimb muscle mass was smaller in both fasting groups. Some groups have shown a maintenance of muscle mass during TRF, but most of these studies combine the fasting with resistance exercise [21,22,23,24], whereas we utilized endurance exercise which may have a less potent anabolic effect on the muscle. Interestingly, it has been shown that TRF decreased IGF-1 and testosterone levels [24]. Decreases in these two anabolic signals could contribute to the smaller muscle mass. Additionally, the activation of AMPK can inhibit the mTOR signaling pathways, also preventing muscle hypertrophy and contributing to the smaller muscle mass. AMPK is activated following an increase in the AMP/ATP ratio [25], and its activity is increased when muscle glycogen is low, both at rest and during exercise. However, AMPK is also believed to increase fatty acid uptake and oxidation [26]. Both fasting [27,28] and exercise activate AMPK acutely in skeletal muscle [29,30,31]. Studies have shown that exercising as low as approximately 60% of max aerobic capacity is sufficient to activate AMPK [30,31,32,33]. While we did not see significant increases in AMPK levels with the different TRF protocols, there was a large variation in AMPK phosphorylation in the TRF groups. Additionally, blood glucose levels remained similar across all groups post exercise without alterations in blood ketones, suggesting that healthy young mice were able to handle the energy stress of the acute exercise bout regardless of the fasting entrainment protocol that they received. When TRF groups are combined, there is a large effect (Cohen’s d = 1.13), suggesting that the stress of fasting in combination with exercise may increase AMPK to a greater extent than exercise in the ad libitum groups. A larger sample would be needed to verify these results and analysis of protein turnover may aid in better understanding the impact of the exercise training and timing of fasting with regard to the acute response of exercise on muscle mass maintenance and AMPK activation.

It is known that both intermittent fasting and exercise training can help improve metabolic flexibility in high-fat diet conditions [34,35]; however, little is known about how these lifestyles affect metabolic flexibility in response to an acute exercise bout in a healthy population. When we examined transcriptional markers of lipid metabolism and glucose metabolism, entrainment with exercise and delayed feeding post exercise yielded lower levels of fatty acid binding protein, hormone sensitive lipase and fatty acid synthase after acute exercise, while there were no differences in the transcription of genes regulating glucose metabolism. Many of the metabolic processes are regulated in a circadian fashion and can be entrained through external stimuli, such as feeding/fasting and exercise [36]. The timing of exercise can also impact the metabolic pathways being utilized [37]. If the entrainment with training and TRF resulted in a phase shift of the clock, this could explain some of the differences observed in the expression of metabolic genes. Additional time points post exercise would be needed to see if the decreases in genes related to lipid metabolism are due to a delay in their expression or a true decrease in the amplitude of their expression, and functional assays such as respiratory exchange ratio (RER) or muscle mitochondrial respiration would be needed to determine if the changes in gene expression yield physiological changes in metabolism in the mice.

To address the changes in genes regulating lipid metabolism, markers of mitochondrial content and biogenesis were measured. While there was a decrease in the mRNA expression of PGC-1α, a marker of mitochondrial biogenesis, in the TRF-delayed group, there was a trend towards an increase in PCG-1α protein expression in the gastrocnemius of the TRF-immediate group compared to the control, and a decrease in TRF-delayed gastrocnemius compared to the TRF-immediate group. PGC-1α gene expression has been shown to acutely increase within 6 h of food removal and return to baseline after 48 h [38]. Additionally, PGC-1α mRNA levels are increased following exercise both acutely [39] and chronically [40], and have previously been shown to increase 24–36 h after exercise [41]. Excess stress placed on the muscle after exercise and the inability to replenish energy stores by delaying feeding after exercise may contribute to the suppressed PGC-1α levels in the TRF-delayed group.

TFAM, a target of PGC-1α, is increased following acute bouts of exercise [42] as well as chronic training [43]. Independently, both fasting and exercise can increase TFAM and PGC1α expression levels [44]. However, one study found that alternate day fasting by itself did not increase TFAM expression, but combining exercise with alternate day fasting resulted in an increase in TFAM expression compared to the control [45]. These mice had access to food immediately post exercise on feeding days, meaning the mice either exercised on a fed state, like our control group, or in a fasted state with immediate access post exercise, like our TRF-immediate group, which could explain the differences that we see in our study.

Although there was a decrease in the transcription of PGC-1α in the delayed group, there were no differences in components of the electron transport chain. Interestingly, researchers looking at exercise at various times of day found that early active phase exercise leads to a diminished transcript related to mitochondrial respiratory function following acute exercise, and an increase in lipid metabolites during activity in the rest phase [37]. In the present study, all animals were exercised in the early active phase. As all animals underwent the same training stimulus and were considered healthy, it suggests that fasting has less of an impact on mitochondrial content in this active healthy population. The exercise training may be a stronger stimulus than the fasting. Long-term fasted state aerobic training has been shown to increase mitochondrial capacity and function in healthy subjects [46]. As all of our animals were trained the same, we cannot assess the effects of exercise training alone; however, much research has already shown exercise-induced adaptations of muscle mitochondria. More work is needed to fully understand and dissect the individual impacts of exercise alone compared to fasting alone and the potential effects of their combination in young healthy populations.

Regardless of the signaling changes in the skeletal muscle, fasting had no effect on performance measured by a run to fatigue. This suggests that the timing of fast does not alter performance in healthy trained mice. In contrast to our findings, Marosi et al. found that mice performed better on a run to exhaustion test during the rest phase after a month of alternate day fasting compared to ad libitum feeding [45]. The differences observed between the studies could be due to the duration of fast. In the present study, mice fasted for a total of 16 h daily, whereas Marosi et al. fasted mice for 24 h [45].

While our study had limitations including the exclusion of a non-trained group, it supports the idea that intermittent fasting in trained individuals is safe and does not impact performance. The existence of benefits other than weight loss suggests that TRF may provide benefits to other populations, not just those seeking weight loss; however, more work is needed to validate the safety and impact of these findings in humans. We demonstrate that when fasting, the immediate consumption of food may offer some benefit; however, more research is needed to better understand the magnitude of these effects.

## Figures and Tables

**Table 1:** Primers used for PCR.

Gene	Forward Primer 5′-3′	Reverse Primer 5′-3′
*Tfam*	TCCCCTCGTCTATCAGTCTTG	GGGCTGCAATTTTCCTAACC
*Pgc1a*	AAGACGGATTGCCCTCATTT	AGTGCTAAGACCGCTGCATT
*Fabp*	GCTGGGAATAGAGTTCGACG	CTTCTCATAAGTCCGAGTGCTC
*Cd36*	GATGTGCAAAACCCAGATGAC	ACAGTGAAGGCTCAAAGATGG
*Crat*	CTGTGGGATGGTGTATGAGC	CTGAGGTTCTGTTTGGCTTTC
*Hsl*	CACAGACCTCTAAATCCCACG	ATATCCGCTCTCCAGTTGAAC
*Pfk*	TGGTGCTGAGGAATGAGAAATG	CCCAGACATCCAGTTCATAGC
*Glut4*	GTAACTTCATTGTCGGCATGG	TGCTCTAAAAGGGAAGGTGTC
*Fasn*	GATGACAGGAGATGGAAGGC	GAGTGAGGCTGGGTTGATAC
*Ldhb*	CCGAAAATTGTGGCCGATAAAG	GCTGTACTTGACGATCTGAGG
*Gapdh*	GTTGTCTCCTGCGACTTCA	TGCTGTAGCCGTATTCA

**Table 2:** Muscle mass.

Muscle	Control	TRF-Immediate	TRF-Delayed
Gastrocnemius (mg)	137.9 ± 2.4	128.3 ± 3.3	126.1 ± 3.4 *
Soleus (mg)	10.3 ± 0.4	9.0 ± 0.2 *	8.9 ± 0.3 *
Plantaris (mg)	18.2 ± 0.5	16.6 ± 0.5	16.3 ± 0.5
Tibialis Anterior (mg)	45.3 ± 1.2	41.2 ± 1.8	40.5 ± 0.9 *
Extensor digitorum longus (mg)	10.8 ± 0.4	9.8 ± 0.4	9.5 ± 0.3
Quadriceps (mg)	101.4 ± 1.8	96.5 ± 3.5	94.2 ± 3.8
Tibia Length (mm)	16.52 ± 0.07	16.60 ± 0.13	16.38 ± 0.16

All data presented as mean ± SEM. An ANOVA was used to measure differences between groups. Significance was set at *p* ≤ 0.05. * significant compared to control.
